# Secondary Syphilis Presenting as Bilateral Simultaneous Papillitis in an Immunocompetent Individual

**DOI:** 10.7759/cureus.28465

**Published:** 2022-08-27

**Authors:** Seth E Buscho, Rhys Ishihara, Praveena K Gupta, Renuka Mopuru

**Affiliations:** 1 Department of Ophthalmology and Visual Sciences, University of Texas Medical Branch, Galveston, USA

**Keywords:** prednisone, papillitis, ocular syphilis, immunocompetent, human immunodeficiency virus

## Abstract

Ocular syphilis is a common presentation for patients with secondary or tertiary syphilis and usually includes posterior uveitis or panuveitis, though a myriad of symptoms have been associated. We report the case of a 58-year-old Caucasian male who presented with fast-progressing vision loss and a new onset of floaters in both eyes. An initial fundus exam revealed only bilateral optic disc edema, and neurological evaluation was negative. Subsequent ophthalmology evaluation in the clinic revealed a ragged retinal pigmented epithelium on optical coherence tomography (OCT) and posterior placoid chorioretinitis, raising suspicion of syphilis. Intravenous penicillin therapy was immediately initiated based on high clinical suspicion of ocular syphilis while awaiting lab confirmation, which was later confirmed as a new syphilis infection. He was subsequently given oral prednisone 48 hours into penicillin therapy for a significant posterior inflammatory response in both his eyes. His visual recovery was drastic due to the timely use of oral steroids. Classical findings such as ragged retinal pigmented epithelium on OCT and posterior placoid chorioretinitis demonstrate strong clinical suspicion of ocular syphilis. Oral prednisone when used timely with penicillin therapy in special situations such as bilateral severe posterior uveitis, panuveitis, or optic neuritis may aid in a faster and smoother visual recovery. A high index of clinical suspicion of ocular syphilis should be maintained in patients with human immunodeficiency virus (HIV) infection presenting with uveitis, posterior placoid morphology, or optic disc edema. Oral prednisone may be an effective adjuvant treatment for immunocompetent patients who mount a strong inflammatory response to ocular syphilis infection.

## Introduction

Syphilis is a sexually transmitted infection caused by the spirochete Treponema pallidum. It is often considered the "great masquerader," as it can mimic a variety of signs and symptoms of other diseases. Since 2001, the incidence of syphilis has increased almost every year in the United States [1]. Typically, syphilis presents in the waxing and waning phases of the disease, and if left untreated, it can progress through four defined stages: primary, secondary, latent, and tertiary syphilis. Primary syphilis is characterized by a chancre at the site of inoculation with similar ocular manifestations such as chancres on the eyelid or conjunctiva [2]. Secondary syphilis often presents as a macular rash with generalized lymphadenopathy as a result of hematogenous dissemination. Several reported ocular signs of secondary syphilis include scleritis, keratitis, anterior uveitis, posterior uveitis, panuveitis, vitritis, chorioretinitis, and necrotizing retinitis. Tertiary syphilis can affect virtually any system of the body and is often devastating to the patient. The most frequently reported ocular manifestation of tertiary syphilis is bilateral diffuse periostitis [3]. While the majority of cases are treated before they progress to late stages, studies suggest rates of neurosyphilis are as high as 3.5% [4]. Since ocular involvement can occur at any stage, identification of the diverse manifestations of ocular syphilis is crucial to the early diagnosis of systemic disease and subsequent treatment of the disease [5,6].

One uncommon presentation of ocular syphilis is acute syphilitic posterior placoid chorioretinitis (ASPPC), with just a few reported cases in immunocompetent patients as it was once thought of as a presentation of the immunocompromised [7,8]. Fundus imaging of a patient with ASPPC reveals one or more irregular, pale yellowish subretinal lesions located at the macula. Another rare presentation of ocular syphilis is optic neuritis/papillitis, which presents as an enhanced swollen optic disc often found in the setting of neurosyphilis and a positive venereal disease research laboratory test (VDRL) [9]. Here, we describe the case of an HIV-positive, immunocompetent male with the coexistence of ASPPC and papillitis who was diagnosed with ocular syphilis and successfully treated with intravenous penicillin and oral prednisone.

## Case presentation

A 58-year-old male with a past medical history of HIV, well controlled on HAART (CD4+ of 978), was admitted to the hospital with progressive vision loss in the left eye for the past three weeks and associated floaters in the last three days. On examination, the patient’s best corrected visual acuity (BCVA) was 20/20 in the right eye (OD) and 20/70 in the left eye (OS). The dilated fundus exam was significant for optic disc edema in both eyes (OU). Magnetic resonance imaging (MRI) of the brain and orbit was negative for any acute findings such as space-occupying lesions, bleeding, or hydrocephalus. Elevated intracranial pressure was ruled out as lumbar puncture revealed normal opening pressure and cerebrospinal fluid (CSF) chemistries and CSF VDRL were also negative. Serology for syphilis was negative three months prior. Pending results from multiple infectious labs, including toxoplasma IgG and fungus culture, the patient was discharged from the hospital for an outpatient follow-up with ophthalmology.

**Figure 1:**
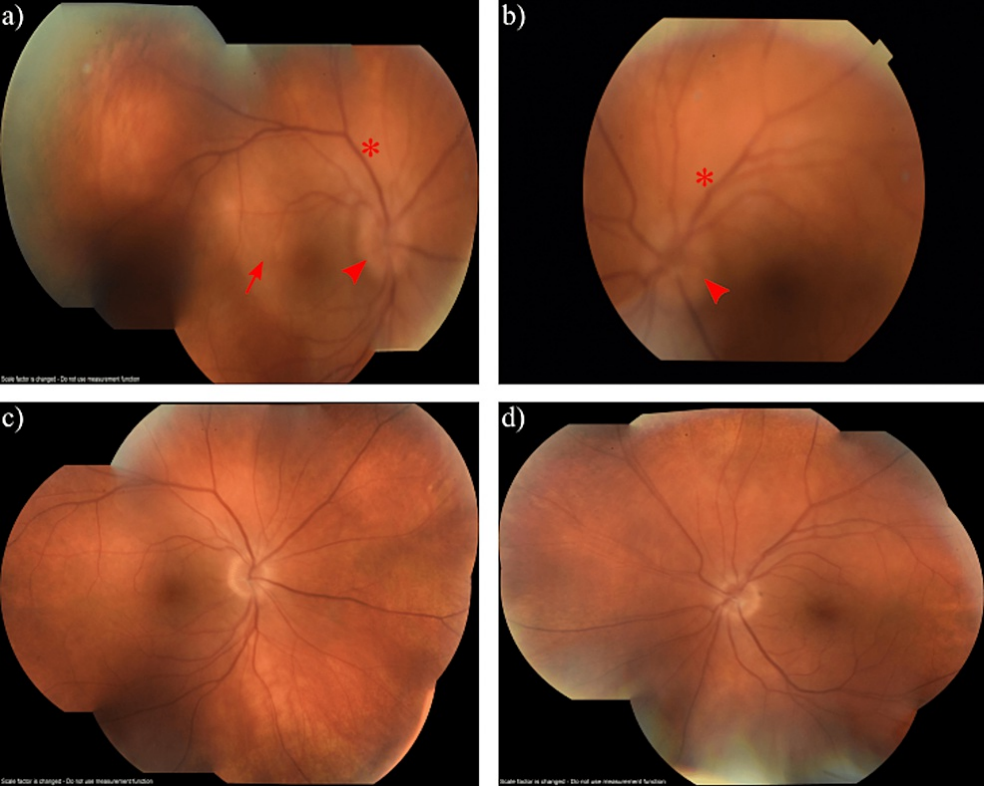
Montage fundus photos showing a round, deep, creamy lesion at the posterior pole of the right eye suggesting ASPPC (arrow), bilateral papillitis (arrowheads), and bilateral venous dilation (asterisks). (a) Before treatment (OD); (b) before treatment (OS); (c) after treatment (OD); (d) after treatment (OS). ASPPC: acute syphilitic posterior placoid chorioretinitis.

**Figure 2:**
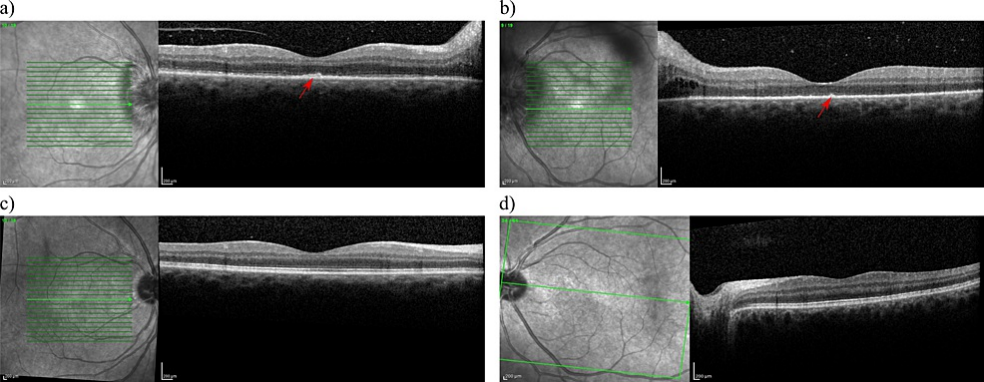
OCT images one week after initial presentation which show granular ragged appearing retinal pigmented epithelium with disruption of the ellipsoid zone (arrow). (a) Before treatment (OD); (b) before treatment (OS); (c) after treatment (OD); (d) after treatment (OS). OCT: optical coherence tomography.

**Figure 3:**
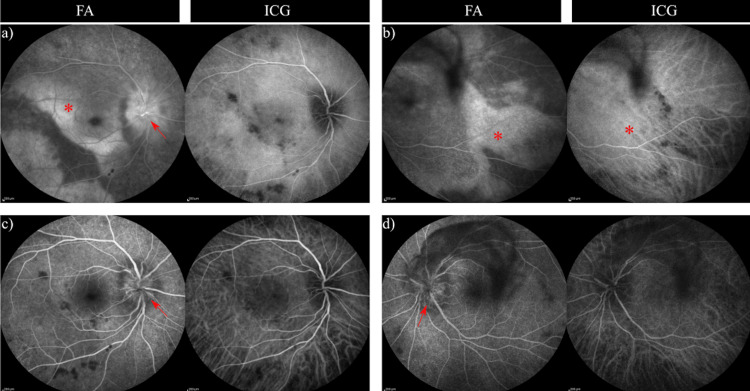
FA and ICG images from one week after initial presentation but before treatment which demonstrate bilateral papillitis (arrow) and posterior placoid morphology which appears as a hyper-fluorescent lesion at the posterior pole (*). (a) Before treatment and early (OD); (b) before treatment and early (OS); (c) before treatment and late (OD); (d) before treatment and late (OS). FA: fluorescein angiography, ICG: indocyanine green.

In his outpatient follow-up one week later, the patient had rapid deterioration of his vision to count fingers (CF) OD and 20/150 OS. At that time, there was a positive relative afferent pupillary defect on the right, and he correctly identified only 1/14 Ishihara plates in both eyes. A dilated fundus exam was significant for a whiteish creamy placoid lesion at the posterior pole of the right eye, bilateral papillitis, and bilateral venous dilation (Figure 1). Further testing with optical coherence tomography (OCT), fluorescein angiography (FA), and indocyanine green angiography (ICG) imaging was conducted. The OCT macula demonstrated disruption of the photoreceptor layer and a ragged appearing hyperreflective retinal pigmented epithelium (Figure 2). FA and ICG were significant for posterior placoid morphology in the macula (Figure 3). At this point, there was high suspicion of ocular syphilis, and a repeat serology for syphilis was sent.

Given the rapidly declining vision, bilateral optic nerve edema, and placoid morphology on FA and ICG, he was empirically started on immediate intravenous penicillin therapy due to strong clinical suspicion of ocular syphilis while awaiting serological confirmation. The diagnosis of syphilis was confirmed with a fluorescent treponemal antibody absorption test (FTA-ABS) three days after the initiation of treatment. On further history, the patient denied any history of genital or oral chancres and he reported no recent history of rashes on his palms or soles of his feet.

Because of the profound bilateral disc edema, chorioretinitis, and vitritis, the patient was started on high-dose oral prednisone 48 hours into his penicillin treatment. The patient completed 14 days of IV penicillin and a quick oral steroid taper, beginning with a 60 mg dose on the first day, followed by a 10 mg decreased dose after each three-day period, and a 5 mg dose for the final three days. One week into his combined prednisone and penicillin treatment, the patient’s vision had improved to 20/400 OD and 20/100 OS. Six weeks after treatment, the patient’s vision returned to 20/20 OU, disc edema was resolved with no evidence of active vitritis, and he correctly identified all 14/14 Ishihara plates in both eyes.

## Discussion

Syphilis is often known as the "great masquerader" because it can affect almost any organ in the body and is present in ways similar to a variety of diseases. Moreover, patients with syphilis can lack distinguishing signs of the disease such as chancre, rash, or gumma, which makes it even more difficult to diagnose and treat. In this case, the patient lacked any systemic manifestations of syphilis, had a negative IgG/IgM test result just three months prior, and was negative for T. pallidum on lumbar puncture. Despite these negative indicators, the unique presentation of bilateral papillitis with ASPPC played a key role in the diagnosis of syphilis.

Since ASPPC is a rare manifestation of ocular syphilis, its pathophysiology is poorly understood and there is disagreement regarding which patient groups are at greatest risk [10]. Some studies suggest infection is the primary stimulus for ASPPC, while other studies report an autoimmune reaction may be the trigger for ASPPC. For example, Neri and Pichi reported a patient who developed a large placoid macular lesion after beginning prednisone treatment for syphilitic uveitis [11]. Another case report documented a patient who resolved his ASPPC following antiviral therapy for HIV and subsequent immune system recovery [12]. On the other hand, Bierowski et al. reported a patient whose floaters and photopsias improved following steroid treatment but prior to penicillin G treatment, indicating an inflammatory mechanism [4,6]. Additionally, Brito et al. found elevated anti-beta2 glycoprotein I antibodies in one patient with ASPPC and Ormaechea et al. reported a patient whose ASPPC relapsed upon decreasing the oral prednisone dose [5,12]. Therefore, high clinical suspicion should be maintained for ocular syphilis in patients who are immunosuppressed as well as immunocompetent, as our patient had a CD4+ of 978 and responded well to steroids, which points towards ASPPC involving an immune response.

The use of corticosteroids is well documented for use in uveitis. However, there is often a fear of using steroids in infectious uveitis. Unfortunately, there is little literature that documents the use of corticosteroids, topical or oral, in the treatment of ocular syphilis. In this case, we utilized oral corticosteroids to treat ocular inflammation and disc edema associated with syphilis as the patient was immunocompetent with a high CD4+ and HIV well-controlled on HAART, indicating an inflammatory component of ASPPC. Although this is just one case report and conclusions cannot be made, oral steroids may be an effective adjuvant treatment for immunocompetent patients on antibiotics who have severe ocular inflammation secondary to syphilis.

For presentations like the current case with bilateral severe inflammatory processes with drastically worsening vision, it is appropriate to treat the patient with empiric antibiotics while awaiting serology confirmation to limit ocular or systemic morbidity. The most common manifestations of ocular syphilis that ophthalmologists should be aware of include posterior uveitis and panuveitis [13]. The less common signs that were presented in this case and led to the diagnosis include bilateral optic nerve neuritis and ASPPC, especially since ASPPC occurs almost exclusively from syphilis etiology [14]. Of note, ocular manifestations can arise early in the course of syphilis, which may make the eye useful in the early detection and treatment of syphilis [15]. In this case, the patient received a true negative syphilis test just three months before the onset of his symptoms, which means he must have contracted syphilis within the past three months. Therefore, early identification of the ophthalmic signs of the disease is critical to prevent blindness and overcome the rapidly increasing incidence of syphilis.

## Conclusions

Syphilis can be notoriously difficult to diagnose given the diverse range of signs it can present with and the variable course of the disease. When examining HIV-positive patients with classic ocular findings such as uveitis or rare findings on multimodal imaging such as bilateral papillitis and posterior placoid morphology, clinicians should maintain suspicion of ocular syphilis. In this case, the patient’s clinical history in combination with the bilateral papillitis and placoid lesions at the posterior pole were key to making the diagnosis of syphilis despite the negative syphilis test result just three months prior. Additionally, oral prednisone in conjunction with appropriate antibiotic therapy may be an effective adjuvant treatment for patients with a severe inflammatory response in the eye. 
